# Evaluation of S1RBD-Specific IgG Antibody Responses following COVID-19 Vaccination in Healthcare Professionals in Cyprus: A Comparative Look between the Vaccines of Pfizer-BioNTech and AstraZeneca

**DOI:** 10.3390/microorganisms10050967

**Published:** 2022-05-04

**Authors:** Elie Deeba, George Krashias, Astero Constantinou, Dana Koptides, Anastasia Lambrianides, Christina Christodoulou

**Affiliations:** 1 Department of Molecular Virology, The Cyprus Institute of Neurology and Genetics, Nicosia 2371, Cyprus; elied@cing.ac.cy (E.D.); astero@cing.ac.cy (A.C.); dankom@cing.ac.cy (D.K.); cchristo@cing.ac.cy (C.C.); 2 Department of Neuroimmunology, The Cyprus Institute of Neurology and Genetics, Nicosia 2371, Cyprus; nancyl@cing.ac.cy

**Keywords:** SARS-CoV-2, vaccine, spike 1 receptor binding protein, IgG antibody

## Abstract

There is an ongoing effort to report data on SARS-CoV-2 antibodies in different individuals. Ninety-seven healthcare workers were enrolled in this study (Pfizer’s BNT162b2, *n* = 52; and AstraZeneca’s ChAdOx1-S, *n* = 45) and S1RBD-specific IgG antibodies were analyzed over time. Both vaccines induced S1RBD-specific antibodies after the second dose. A significant increase in S1RBD-specific IgG median levels 3 weeks following the second dose was detected (BNT162b2, 118.0 BAU/mL to 2018.0 BAU/mL; ChAdOx1-S, 38.1 BAU/mL to 182.1 BAU/mL). At 3 months post the second dose, a significant decrease in S1RBD-specific IgG median levels was also evident (BNT162b2, 415.6 BAU/mL, ChAdOx1-S, 84.7 BAU/mL). The elimination rate of these antibodies was faster in BNT162b2- rather than ChAdOx1-S- vaccinated individuals. A booster dose induced a significant increase in the S1RBD-specific IgG median levels (BNT162b2, 1823.0 BAU/mL; ChAdOx1-S, 656.8 BAU/mL). This study is the first of its kind to characterize S1RBD-specific IgG antibody responses in vaccinated healthcare workers in Cyprus. While the positivity for S1RBD-specific antibodies was maintained 3 months after the second vaccine dose, the level of these antibodies waned over the same period, indicating the importance of a booster vaccination. The results herein could complement the public health policies regarding the immunization schedule for COVID-19.

## 1. Introduction

Severe acute respiratory syndrome coronavirus 2 (SARS-CoV-2), the causative agent for Coronavirus disease 2019 (COVID-19), has claimed over 6 million lives globally (April 2022) [1].

The unprecedented global effort to develop vaccines against SARS-CoV-2 has led to the emergency approval of various vaccines around the world [2]. As of March 2022, a total of more than 11 billion vaccine doses have been administered worldwide [1]. Irrespective of the technology utilized, all of the approved COVID-19 vaccines, including the leading SARS-CoV-2 vaccines Pfizer-BioNTech’s BNT162b2 (Comirnaty) and AstraZeneca’s ChAdOx1-S nCoV-19/AZD1222 (Vaxzevria), aim to induce neutralizing antibodies against the spike (S) glycoprotein of SARS-CoV-2 [2]. As shown in a number of studies these neutralizing antibodies are considered to be a reliable biomarker for the correlate of protection against SARS-CoV-2 infection [3,4,5,6,7]. The importance of vaccination is further highlighted in a recent study indicating that approximately 470,000 lives have been saved among those aged 60 years and over since the start of the COVID-19 vaccination roll-out in 33 countries across the WHO European Region [8].

Evaluating SARS-CoV-2-specific antibody responses following COVID-19 vaccination is of primary importance, since it allows to establish the magnitude and persistence of humoral immunity over time. COVID-19 vaccination in Cyprus began on 28 December 2020. Amongst the first to be vaccinated were healthcare workers that are at increased risk of being infected with SARS-CoV-2 [9]. Obtaining information related to the kinetics of SARS-CoV-2 -specific antibodies following vaccination is important to develop strategies to maximize the impact of the approved vaccines among healthcare workers. To the best of our knowledge, the study herein is the first to analyze the persistence of antibodies in healthcare workers in the Republic of Cyprus following vaccination with Pfizer-BioNTech’s BNT162b2 and AstraZeneca’s ChAdOx1-S nCoV-19.

## 2. Materials and Methods

### 2.1. Ethical Approval and Subject Recruitment

This study was approved by the Cyprus National Bioethics Committee (ΕΕΒΚ/ΕΠ/2020/23). The study inclusion criteria were >18 years of age, and who had tested negative for SARS-CoV-2 infection prior to each administered vaccine dose. Study participants were from the staff (doctors, nurses, and researchers) of the Cyprus Institute of Neurology and Genetics (CING). Upon enrolment, all participants provided information related to previous history of COVID-19, i.e., either RT-PCR or rapid antigen test results for SARS-CoV-2 detection, as well as the type of vaccination. Note that any volunteer’s positive history of SARS-CoV-2 infection at any point during the study was reason for exclusion from analysis. All participants completed and signed an informed consent form.

### 2.2. Study Population and Sample Collection

A total of 52 BNT162b2 -vaccinated participants (PfVac) and 45 ChAdOx1-S -vaccinated participants (AzVac) signed up for the study. Blood samples were collected at the following time points: T1; just before the second vaccination dose, i.e., 3 weeks after the first BNT162b2 vaccination dose and 12 weeks after the first ChAdOx1-S vaccination dose, T2; three weeks after the second vaccination dose for both types of vaccine, T3; 3 months after the second vaccination dose for both types, and finally T4, three months after the third vaccination dose (booster dose) for both types. Participants were recruited as soon as vaccination campaigns started, i.e., February 2021 for BNT162b2 and April 2021 for ChAdOx1-S. Note that 21 PfVac participants gave a sample at T4 and were vaccinated with BNT162b2 as a booster dose, 5 AzVac participants gave a sample at T4 with the mRNA-1273 (Moderna’s Spikevax COVID-19 vaccine) as their booster dose, and 20 AzVac participants gave a sample at T4 with the ChAdOx1-S as their booster dose. The characteristics for all participants are summarized in Table 1.

Blood samples were collected in tubes containing clotting activators at the COVID-19 sampling unit of the CING. Following blood collection, samples were centrifuged for 10 min at 500× *g* at 20 °C to obtain cell-free serum. Serum was stored at −20 °C until analysis.

### 2.3. Sample Processing and Analysis

Serum obtained from the two groups of the study was used to quantify the level of Anti-S1RBD IgG antibodies. The quantification was performed using the ABBOTT SARS-CoV-2 IgG II Quant assay (REF# 6S60-22) on an ABBOTT ARCHITECT i1000SR instrument. The assay is an automated, two-step chemiluminescent microparticle immunoassay used for qualitative and quantitative determination of IgG antibodies against the Spike1 receptor-binding domain (S1RBD) of the SARS-CoV-2 from human serum and plasma. The SARS-CoV-2 IgG II Quant calibrator package (REF# 6S60-02) and the SARS-CoV-2 IgG II Quant control package (REF# 6S60-12) were run on the instrument prior to sample analysis. According to the manufacturer, the cut-off is set at 50.0 AU/mL, and the analytical measuring interval is set between 21.0 (limit of quantification) and 40,000.0 AU/mL (upper limit of quantification). Additional information on performance characteristics of the assay can be found in the manufacturer’s manual. Based on the recommendations of the National Institute of Biological Standards and Control (NIBSC) and WHO, the concentrations were converted into Binding antibody units per mL (BAU/mL) through multiplying AU/mL by a factor of 0.142. The corresponding cut-off value becomes 7.1 BAU/mL.

The rate of change (RC) between 3 weeks from the second dose (T2) and 3 months from the second dose (T3) was calculated as the ratio between the difference in antibody levels at the time intervals over the length of the time interval (T3 − T2 = 71 days). The percentage change per month (%change/mo) and per year (%change/yr) were also calculated to facilitate comparison with other studies. Note that a decrease in RC or %change is presented as a negative number.

### 2.4. Statistical Analysis

The Wilcoxon signed-rank test was employed to evaluate the significance in the change between each consecutive sampling from the participants. The Mann-Whitney U test was used to evaluate significance in RC between the different vaccine regimens. The GraphPad Prism v8.00 for Windows software program was used to perform the statistical analyses (GraphPad Software, La Jolla, CA, USA).

## 3. Results

### 3.1. Kinetic Change in Anti-S1RBD IgG Antibodies during the Initial Vaccination Regimen with BNT162b2 and ChAdOx1-S

Overall, both vaccines were efficient at inducing S1RBD-specific antibodies following the initial dose in the majority of vaccinated individuals. In detail, 3 weeks after the initial vaccination (T1) 94.2% in the PfVac group and 97.8% in the AzVac group had detectable antibodies against S1RBD of SARS-CoV-2. Following the second dose both vaccines were 100% effective at inducing S1RBD-specific antibodies.

**Figure 1:**
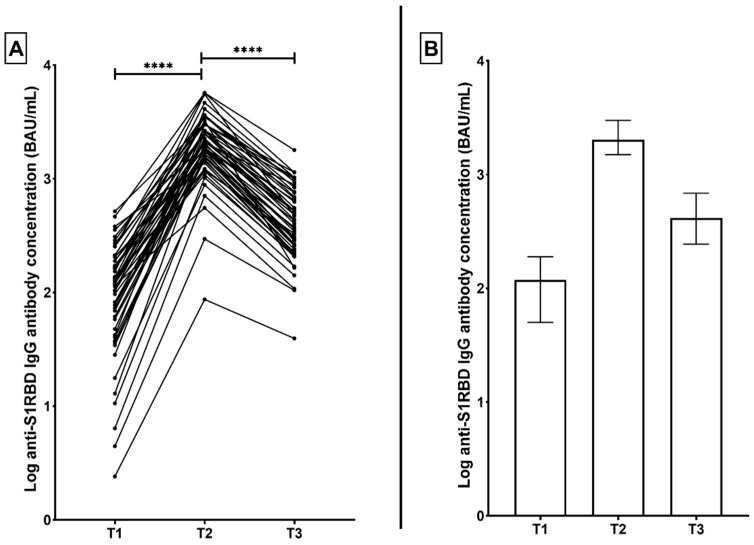
(**A**) Correlation graph representing the change in anti-S1RBD IgG antibody concentrations in BNT162b2 -vaccinated participants (PfVac) across three time points; 3 weeks after the first vaccination dose (T1), 3 weeks after the second dose (T2), and 3 months after the second dose (T3). (**B**) corresponding distribution graph with bars representing median and interquartile range. **** *p* < 0.0001.

Quantification of antibodies against the S1RBD of SARS-CoV-2 revealed that in the PfVac group, anti-S1RBD IgG antibodies were measured at a median (interquartile range) of 118.0 BAU/mL (50.3 to 190.3) 3 weeks after the initial vaccination (T1). The concentration of S1RBD-specific antibodies significantly increased after the second vaccination dose (T2), to 2018.0 BAU/mL (1496.0 to 2999.0) (*p* < 0.0001). At 3 months after the second dose (T3), the concentration was significantly reduced to 415.6 BAU/mL (244.9 to 686.5) (*p* < 0.0001) while still retaining positivity in all volunteers (Figure 1).

**Figure 2:**
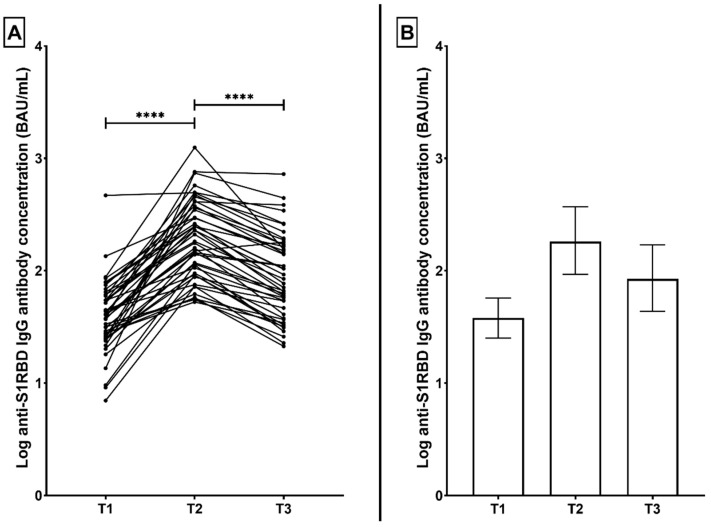
(**A**) Correlation graph representing the change in anti-S1RBD IgG antibody concentrations in ChAdOx1-S -vaccinated participants (AzVac) across three time points; 12 weeks after the first vaccination dose (T1), 3 weeks after the second dose (T2), and 3 months after the second dose (T3). (**B**) corresponding distribution graph with bars representing median and interquartile range. **** *p* < 0.0001.

Similarly, in the AzVac group, anti-S1RBD IgG antibodies were measured at a median (interquartile range) of 38.1 BAU/mL (25.2 to 57.2) following 12 weeks after the initial vaccination dose (T1). The concentration significantly increased at T2 to 182.1 BAU/mL (93.1 to 370.3) (*p* < 0.0001). At T3, the concentration had significantly dropped to 84.7 BAU/mL (43.7 to 170.4) (*p* < 0.0001) while still retaining positivity in all volunteers (Figure 2).

### 3.2. The Rate of Anti-S1RBD IgG Antibody Clearance between 3 Weeks and 3 Months Post Second Vaccination Dose

The rate of change was used to evaluate how quickly S1RBD IgG antibodies decreased after the second dose. The RC is thus calculated as the decrease in the level of antibody per day (BAU/mL · day). The median RC between 3 weeks (T2) and 3 months (T3) from the second dose was significantly higher in the PfVac group (−21.9 BAU/mL · day [−29.4 to −16.8]) than in the AzVac group (−1.2 BAU/mL · day [−2.4 to −0.5]) (*p* < 0.0001) (Table 2).

### 3.3. Kinetic Change in Anti-S1RBD IgG Antibodies following Booster Doses with Various Vaccine Types

Around half of the participants from each group agreed to return for a fourth time, i.e., three months after the booster dose (T4). Therefore, the kinetics analysis that includes T4 was performed separately, resulting in three separate groups: PfVac with BNT162b2 (PfVac-Pf) (*n* = 21), AzVac with ChAdOx1-S (AzVac-Az) (*n* = 20), and AzVac with mRNA-1273 (AzVac-Md) (*n* = 5).

**Figure 3:**
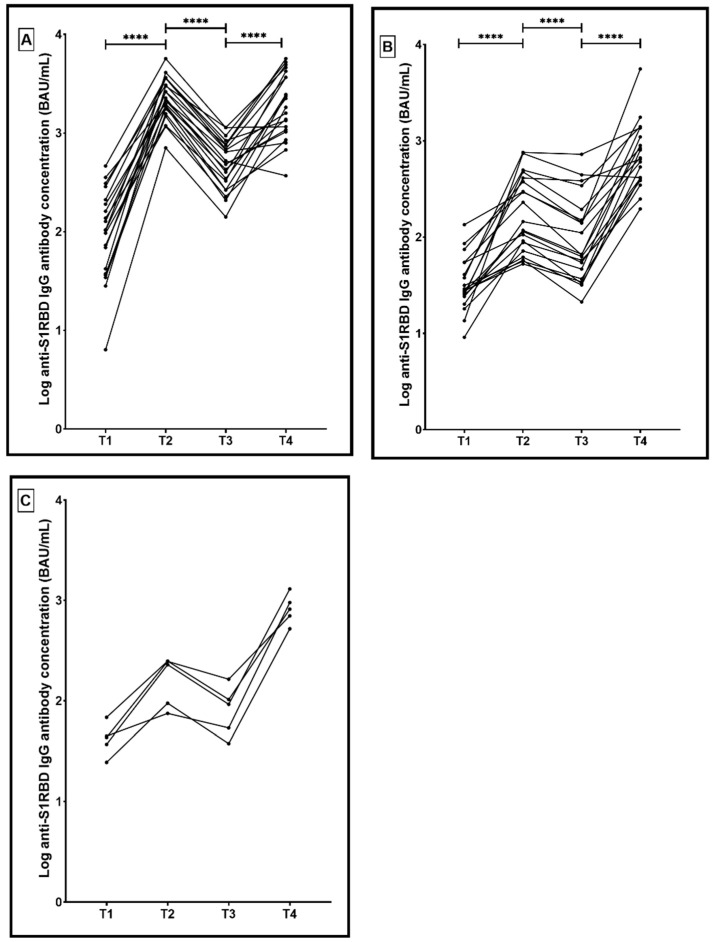
(**A**) Correlation graph representing the change in anti-S1RBD IgG antibody concentrations in participants vaccinated with three doses of BNT162b2 (PfVac-Pf) across four time points; 3 weeks after the first vaccination dose (T1), 3 weeks after the second dose (T2), 3 months after the second dose (T3), and 3 months after the booster dose. (**B**) Correlation graph representing the change in anti-S1RBD IgG antibody concentrations in participants vaccinated with three doses of ChAdOx1-S (AzVac-Az) across four time points; 12 weeks after the first vaccination dose (T1), 3 weeks after the second dose (T2), 3 months after the second dose (T3), and 3 months after the booster dose. (**C**) Correlation graph representing the change in anti-S1RBD IgG antibody concentrations in participants vaccinated with two doses of ChAdOx1-S and one dose of mRNA-1273 (AzVac-Md) across four time points; 12 weeks after the first vaccination dose (T1), 3 weeks after the second dose (T2), 3 months after the second dose (T3), and 3 months after the booster dose. **** *p* < 0.0001.

Anti-S1RBD IgG antibodies significantly increased from 489.3 BAU/mL (297.3 to 761.8) at T3 to 1823.0 BAU/mL (1055.0 to 3952.0) at T4 (*p* < 0.0001) in the PfVac-Pf group (Figure 3A). Similarly, antibody levels significantly increased from 65.8 BAU/mL (39.3 to 183.2) at T3 to 656.8 BAU/mL (407.6 to 1298.0) at T4 (*p* < 0.0001) in the AzVac-Az group (Figure 3B). Antibody levels increased from 92.4 BAU/mL (45.8 to 133.6) at T3 to 819.5 BAU/mL (611.8 to 1127.0) at T4 (*p* = 0.6) in the AzVac-Md group (Figure 3C). Additionally, the median antibody level of anti-S1RBD IgG at T4 was significantly higher in the PfVac-Pf group compared to either the AzVac-Az or AzVac-Md groups (*p* = 0.0002).

## 4. Discussion

The CING is the national leading institute in the Republic of Cyprus dealing with all autoimmune, genetic, and neurological diseases, and thus welcoming hundreds of immunocompromised patients on a daily basis. Therefore, vaccination of its staff was of high priority considering healthcare professionals worldwide were among the first to be given the option of vaccination. As a result, the present study was conducted in order to measure the persistence and kinetics of anti-S1RBD IgG antibodies in the different individuals working at CING.

Regardless of vaccine type, all participants presented with positive levels of antibodies 3 weeks after the second vaccination dose, which remained positive 3 months after the second vaccine. These results agree with other studies showing that antibody levels can indeed last for months following vaccination [10,11,12,13,14]. Additionally, our findings are in line with other similar international studies, highlighting the decline of S1RBD-specific antibodies over time [10,11,12,13,14,15]. Nevertheless, the immunogenic capacity of the antibodies present needs to be investigated further in order to determine their effectiveness in case of an infection with any of the emerging SARS-CoV-2 variants of concern.

The decrease in anti-S1RBD IgG levels was expected, based on not only the current working knowledge in vaccinology [16,17], but also specifically, on recent SARS-CoV-2-centered studies [14,15,18,19,20]. More importantly, it is worth noting that, in these latter studies as well as the current study, the observed rate of antibody clearance is significantly higher than that from other established vaccines, such as the MMR vaccine [16,17]. Our results show a 403% and 270% annual decrease in antibody levels from BNT162b2 and ChAdOx1-S vaccination types respectively as opposed to a 5–10% annual decrease for the MMR vaccine [16,17]. It is possible that the rapid decrease in antibodies through time as compared to other established vaccines, especially comparable ones that do not contain adjuvants, may be attributed to the nature of the vaccine itself, i.e., a relatively new technology that uses RNA encoding a specific epitope from the whole virus. This is not to say, of course, that the vaccine is not effective in its current form, but rather does not present a long-term solution to the pandemic. We need to take into consideration the urgency with which the vaccine was created and produced which offered fast-tracked approval by the relevant authorities and immediate global distribution.

Calculating the rate of change in antibody levels after the second vaccination dose allowed for a normalized comparison between the different vaccine regimens. Our results suggest that the overall elimination rate of IgG antibodies is faster in PfVac participants than in AzVac participants. On the other hand, another study performed in Greece, suggested the opposite, i.e., the elimination rate of IgG antibodies was found to be faster in participants vaccinated with ChAdOx1-S than in those vaccinated with BNT162b2 [14]. More specifically, our results showed the RC ratio of BNT162b2 to ChAdOx1-S at 17.9:1 as compared to 1:2.6 in the other study [14]. Independent data around ChAdOx1-S and its comparison with other SARS-CoV-2 vaccines are scarce. Therefore, differing comparative studies are essential in understanding the heterogeneity of the immune response across individuals as well as across different vaccination regimens.

Due to this waning humoral immunity observed worldwide as well as the possible reduced efficacy against emerging variants of concern, health authorities have recommended a booster dose to be administered 6 months after the initial vaccination series, regardless of SARS-CoV-2 infection status [21]. Consequently, participants were asked to provide one further sample 3 months after the booster dose. Our results show a marked increase in antibody levels regardless of the booster type administered, as similarly observed in other studies [12,22,23]. Several studies have shown that the relative effectiveness of having a booster dose regardless of the primary vaccination regimen continues to outweigh the risks of having severe symptoms upon infection [21,22,24]. It is vital for us to continue studying the kinetics of the antibodies produced across time, as well as the antibody effectiveness against other SARS-CoV-2 variants. Such studies would allow for further understanding of immune response kinetics, ultimately allowing for quicker global responses against future emerging variants. In our case, direct comparison at 3 months after the booster dose between the different groups showed significantly higher antibody levels in the PfVac-Pf group than in the AzVac-Pf and AzVac-Md groups combined. Our observation appears to contradict other studies that favor heterologous vaccination regimens, i.e., ChAdOx1-S/BNT162b2 or ChAdOx1-S/mRNA-1273, as opposed to homologous vaccination regimens, i.e., BNT162b2/BNT162b2 [25,26]. Therefore, we approach this comparison with caution, taking into consideration that several limitations and/or variables may have contributed to such outcomes. Examples of the limitations/variables include, but are not limited to, the small number of samples collected at the last time point, the time differences between the initial vaccination regimens, and the variability in the rates of antibody clearance between the vaccination regimens, as well as between individuals themselves.

One limitation of the study as a whole is the limited sample size, which may affect studying confounding variables that affect individual immune systems, such as autoimmune disorders and/or different comorbidities. Due to the nature of the study, we were confined to a certain age range corresponding to the institute’s health professionals, as well as the type of vaccinations studied, since BNT162b2 and ChAdOx1-S are the most widely administered types in the Republic of Cyprus. Additionally, the participant dropout after the booster dose necessitated the creation of further subgroups, which means that such results should be approached with caution and not be considered as wholly representative of the general population. Future studies can incorporate data about SARS-CoV-2 infections after the booster doses, thus allowing for a deeper insight into the effectiveness of the vaccines/booster as well as the duration of protection. It is of equal importance for similar studies to be conducted examining the immunogenicity of other SARS-CoV-2 vaccines being administered worldwide and compare their efficacy against each other.

## 5. Conclusions

The current study followed the persistence of antibodies in healthcare workers in the Republic of Cyprus following vaccination with BNT162b2 and ChAdOx1-S as well as after the booster dose. We were able to show that there is a relatively rapid decrease in S1RBD IgG antibodies following the second vaccination dose from either vaccine type, which was then circumvented by the booster dose allowing for a significantly higher antibody level even after 3 months from the booster as compared to 3 months from the second dose of either vaccine type. Our results may aid the global effort in understanding antibody kinetics across different individuals and across different vaccination regimens. This may also help in better informing public health policies regarding the timing of booster vaccine administration, effectiveness of antibodies produced, as well as considerations towards new emerging variants of concern.

## Figures and Tables

**Table 1:** Characteristics of study participants.

Participants Vaccinated with	Total Number of Participants (*n*)	Age (Mean ± SD)	Sex (Male/Female)
BNT162b2 (PfVac)	52	46.7 ± 12.9	21/31
ChAdOx1-S (AzVac)	45	40.1 ± 11.4	16/29

**Table 2:** Rate of change (RC) and percentage change per month (%change/mo) and percentage change per year (%change/yr) of anti-S1RBD IgG antibodies between 3 weeks after the second dose and 3 months after the second dose in participants vaccinated with BNT162b2 and ChAdOx1-S.

Participants Vaccinated with	Median RC (Interquartile Range)	Median %Change/mo (Interquartile Range)	Median %Change/yr (Interquartile Range)
BNT162b2 (PfVac)	−21.9 BAU/mL · day (−29.4 to −16.8)	−33.6% (−34.7 to −31.7)	−403.5% (−416.5 to −380.1)
ChAdOx1-S (AzVac)	−1.2 BAU/mL · day (−2.4 to −0.5)	−22.5% (−26.5 to −14.8)	−270.0% (−318 to −177.4)
*p* value	*p* < 0.0001		

## Data Availability

The data presented in this study are available on request from the corresponding author. The data are not publicly available due to privacy and ethical reasons.

## References

[1] WHO Coronavirus (COVID-19) Dashboard|WHO Coronavirus (COVID-19) Dashboard with Vaccination Data. https://covid19.who.int/.

[2] Heinz F.X., Stiasny K. (2021). Distinguishing Features of Current COVID-19 Vaccines: Knowns and Unknowns of Antigen Presentation and Modes of Action. NPJ Vaccines.

[3] Khoury D.S., Cromer D., Reynaldi A., Schlub T.E., Wheatley A.K., Juno J.A., Subbarao K., Kent S.J., Triccas J.A., Davenport M.P. (2021). Neutralizing Antibody Levels Are Highly Predictive of Immune Protection from Symptomatic SARS-CoV-2 Infection. Nat. Med..

[4] McMahan K., Yu J., Mercado N.B., Loos C., Tostanoski L.H., Chandrashekar A., Liu J., Peter L., Atyeo C., Zhu A. (2020). Correlates of Protection against SARS-CoV-2 in Rhesus Macaques. Nature.

[5] Corbett K.S., Nason M.C., Flach B., Gagne M., O’Connell S., Johnston T.S., Shah S.N., Edara V.V., Floyd K., Lai L. (2021). Immune Correlates of Protection by MRNA-1273 Vaccine against SARS-CoV-2 in Nonhuman Primates. Science.

[6] Earle K.A., Ambrosino D.M., Fiore-Gartland A., Goldblatt D., Gilbert P.B., Siber G.R., Dull P., Plotkin S.A. (2021). Evidence for Antibody as a Protective Correlate for COVID-19 Vaccines. Vaccine.

[7] Kustin T., Harel N., Finkel U., Perchik S., Harari S., Tahor M., Caspi I., Levy R., Leshchinsky M., Ken Dror S. (2021). Evidence for Increased Breakthrough Rates of SARS-CoV-2 Variants of Concern in BNT162b2-MRNA-Vaccinated Individuals. Nat. Med..

[8] Meslé M.M., Brown J., Mook P., Hagan J., Pastore R., Bundle N., Spiteri G., Ravasi G., Nicolay N., Andrews N. (2021). Estimated Number of Deaths Directly Averted in People 60 Years and Older as a Result of COVID-19 Vaccination in the WHO European Region, December 2020 to November 2021. Eur. Commun. Dis. Bull..

[9] Nguyen L.H., Drew D.A., Graham M.S., Joshi A.D., Guo C.G., Ma W., Mehta R.S., Warner E.T., Sikavi D.R., Lo C.H. (2020). Risk of COVID-19 among Front-Line Health-Care Workers and the General Community: A Prospective Cohort Study. Lancet Public Health.

[10] Choi J.H., Kim Y.R., Heo S.T., Oh H., Kim M., Lee H.R., Yoo J.R. (2022). Healthcare Workers in South Korea Maintain a SARS-CoV-2 Antibody Response Six Months after Receiving a Second Dose of the BNT162b2 MRNA Vaccine. Front. Immunol..

[11] Wang R.C., Murphy C.E., Kornblith A.E., Hohenstein N.A., Carter C.M., Wong A.H.K., Kurtz T., Kohn M.A. (2022). SARS COV-2 Anti-Nucleocapsid and Anti-Spike Antibodies in an Emergency Department Healthcare Worker Cohort: September 2020–April 2021. Am. J. Emerg. Med..

[12] Edelstein M., Beiruti K.W., Ben-Amram H., Bar-Zeev N., Sussan C., Asulin H., Strauss D., Bathish Y., Zarka S., Abu Jabal K. (2022). Antibody-Mediated Immunogenicity against SARS-CoV-2 Following Priming, Boosting and Hybrid Immunity: Insights from 11 Months of Follow-up of a Healthcare Worker Cohort in Israel, December 2020–October 2021. Clin. Infect. Dis..

[13] Matusali G., Sberna G., Meschi S., Gramigna G., Colavita F., Lapa D., Francalancia M., Bettini A., Capobianchi M.R., Puro V. (2022). Differential Dynamics of SARS-CoV-2 Binding and Functional Antibodies upon BNT162b2 Vaccine: A 6-Month Follow-Up. Viruses.

[14] Terpos E., Karalis V., Ntanasis-Stathopoulos I., Evangelakou Z., Gavriatopoulou M., Manola M.S., Malandrakis P., Gianniou D.D., Kastritis E., Trougakos I.P. (2022). Comparison of Neutralizing Antibody Responses at 6 Months Post Vaccination with BNT162b2 and AZD1222. Biomedicines.

[15] Kim J.Y., Lim S.Y., Park S., Kwon J.-S., Bae S., Park J.Y., Cha H.H., Seo M.H., Lee H.J., Lee N. (2022). Immune Responses to the ChAdOx1 NCoV-19 and BNT162b2 Vaccines and to Natural Coronavirus Disease 2019 Infections Over a 3-Month Period. J. Infect. Dis..

[16] Davidkin I., Jokinen S., Broman M., Leinikki P., Peltola H. (2008). Persistence of Measles, Mumps, and Rubella Antibodies in an MMR-Vaccinated Cohort: A 20-Year Follow-Up. J. Infect. Dis..

[17] Seagle E.E., Bednarczyk R.A., Hill T., Fiebelkorn A.P., Hickman C.J., Icenogle J.P., Belongia E.A., McLean H.Q. (2018). Measles, Mumps, and Rubella Antibody Patterns of Persistence and Rate of Decline Following the Second Dose of the MMR Vaccine. Vaccine.

[18] Mishra S.K., Pradhan S.K., Pati S., Sahu S., Nanda R.K. (2021). Waning of Anti-Spike Antibodies in AZD1222 (ChAdOx1) Vaccinated Healthcare Providers: A Prospective Longitudinal Study. Cureus.

[19] Levin E.G., Lustig Y., Cohen C., Fluss R., Indenbaum V., Amit S., Doolman R., Asraf K., Mendelson E., Ziv A. (2021). Waning Immune Humoral Response to BNT162b2 Covid-19 Vaccine over 6 Months. N. Engl. J. Med..

[20] Kwok S.L.L., Cheng S.M.S., Leung J.N.S., Leung K., Lee C.K., Peiris J.S.M., Wu J.T. (2022). Waning Antibody Levels after COVID-19 Vaccination with MRNA Comirnaty and Inactivated CoronaVac Vaccines in Blood Donors, Hong Kong, April 2020 to October 2021. Eurosurveillance.

[21] Joint Statement from HHS Public Health and Medical Experts on COVID-19 Booster Shots|CDC Online Newsroom|CDC. https://www.cdc.gov/medipa/releases/2021/s0818-covid-19-booster-shots.html.

[22] Romero-Ibarguengoitia M.E., Rivera-Salinas D., Hernández-Ruíz Y.G., Armendariz-Vázquez A.G., González-Cantú A., Barco-Flores I.A., González-Facio R., Montelongo-Cruz L.P., del Rio-Parra G.F., Garza-Herrera M.R. (2022). Effect of the Third Dose of BNT162b2 Vaccine on Quantitative SARS-CoV-2 Spike 1-2 IgG Antibody Titers in Healthcare Personnel. PLoS ONE.

[23] Flaxman A., Marchevsky N.G., Jenkin D., Aboagye J., Aley P.K., Angus B., Belij-Rammerstorfer S., Bibi S., Bittaye M., Cappuccini F. (2021). Reactogenicity and Immunogenicity after a Late Second Dose or a Third Dose of ChAdOx1 NCoV-19 in the UK: A Substudy of Two Randomised Controlled Trials (COV001 and COV002). Lancet.

[24] Andrews N., Stowe J., Kirsebom F., Toffa S., Sachdeva R., Gower C., Ramsay M., Bernal J.L. (2022). Effectiveness of COVID-19 Booster Vaccines against COVID-19-Related Symptoms, Hospitalization and Death in England. Nat. Med..

[25] Mayr F.B., Talisa V.B., Shaikh O., Yende S., Butt A.A. (2022). Effectiveness of Homologous or Heterologous Covid-19 Boosters in Veterans. N. Engl. J. Med..

[26] Nguyen T.T., Quach T.H.T., Tran T.M., Phuoc H.N., Nguyen H.T., Vo T.K., van Vo G. (2022). Reactogenicity and Immunogenicity of Heterologous Prime-Boost Immunization with COVID-19 Vaccine. Biomed. Pharmacother..

